# Deficiency in infiltration of CD8/CD3 positive lymphocytes and macrophages plays a role in lip carcinogenesis: an immunohistochemical study

**DOI:** 10.1016/j.bjorl.2023.101379

**Published:** 2023-12-19

**Authors:** Iana Aragão Magalhães, Gabriella Alves Juliao Costa, Marcela Maria Fontes Borges, Anna Clara Aragão Matos Carlos, Karine Cestaro Mesquita, Larissa Mont´Alverne de Arruda, Maria do Perpétuo Socorro Saldanha Cunha, Fabrício Bitu Sousa, Paulo Goberlânio de Barros Silva

**Affiliations:** a Unichristus, Departamento de Odontologia, Fortaleza, CE, Brazil; b Universidade Federal do Ceará, Faculdade de Farmácia, Odontologia e Enfermagem, Departamento de Clínica Dentária, Divisão de Patologia Oral, Fortaleza, CE, Brazil; c Hospital Haroldo Juaçaba, Instituto Cearense do Câncer, Fortaleza, CE, Brazil

**Keywords:** Lip neoplasms, Tumor microenvironment, Lymphocytes, Tumor-infiltrating

## Abstract

**Objective:**

To evaluate the immunoexpression profile for CD8, CD3, CD20 and CD68 in the process and carcinogenesis of Carcinoma of the vermilion lip.

**Methods:**

Average cell count with positive expression for CD3, CD8, CD20 and CD68. The CD8/CD3 ratio calculated in the region was based on the percentage of positive cells in a total of malignant cells. Kruska-Wallis/Dunn, Mann-Whitney and Spearman correlation tests (SPSS, *p* < 0.05) were used.

**Results:**

In the Aquitic Cheilitis samples, there was an increase in intraepithelial CD8+ and CD68+. In LSCCs, there was an increase in peritumoral and intratumoral CD3+, CD8+, CD20+ and CD68+ cells. In peritumoral LSCC, CD3+ and CD8+ showed a direct correlation (*p* = 0.004), and CD68+ and CD8+ (*p* = 0.017). In the intraepithelial region, CD8+ correlated with CD20+ (*p* = 0.014) and CD68+ (*p* = 0.013). In the CAs, CD3 (*p* < 0.001) and CD8 (*p* = 0.025) correlated intraepithelial and subepithelial. In LSCC CD3+ (*p* = 0.002), CD8+ (*p* = 0.001) and CD68+ (*p* = 0.030) had intra and peritumoral correlation.

**Conclusion:**

CD68+ is the first interacting cell with the greatest capacity to migrate to the tumor and interact with CD3, CD8 and CD20. Apparently, CD20 affects perineural invasion.

**Level of evidence:**

Level 2.

## Highlights

• Macrophages (CD68+) migrate into the tumor and interact with CD3, CD8 and CD20.
• CD20 affects perineural invasion and histological gradation.
• More aggressive tumors have lower amounts of CD20.

## Introduction

Carcinoma of the vermilion lip encompasses a set of malignant neoplasms that develop on the lip due to excessive sun exposure over the years.^1^ Among the most frequent tumors, Squamous Cell Carcinoma is the most common histological type at this anatomical site, and UV light is the leading risk factor. However, unlike other tumors located intraorally, its prognosis is considerably good. Due to this fact, studies have been developed to understand the involvement of the tumor microenvironment in controlling the proliferation and the process of oral canciongenesis.^1^, ^2^

The tumor microenvironment comprises all the cellular and non-cellular elements in the tumor-supporting stroma and may present several types of inflammatory cells. Faced with cytokines and growth factors, the interaction of tumor and non-tumor cells can influence these tumors' initiation, progression, metastasis, and even drug resistance. The primary cells present in tumor microenvironment are T-lymphocytes that significantly impact the prognosis of head and neck cancers.^3^, ^4^ Macrophages are also present and seem to show good predictive value as a prognostic marker of survival in cancer patients.^5^ Furthermore, B-lymphocytes play a crucial role in regulating immune responses involved in inflammation and autoimmunity and the recent association with cancer.^6^ A positive correlation between CD20 + B-cell infiltration and vascular endothelial growth factor expression has aggravated angiogenesis and augmented tumor progression.^7^

The evaluation of tumor microenvironment in oral carcinogenesis is considered a recent field of research. Studies have shown that the communication between epithelial cells and tumor microenvironment interferes in processes ranging from tumor initiation and neoplastic progression to metastasis and has a high relationship with prognosis and therapeutic response.^8^

Although the immune-inflammatory profile of head and neck cancers is already widely described, lip cancer, which has the same histological type and better prognosis, has been poorly associated with this context. Since the inflammatory profile may directly impact the prognosis of head and neck tumors, the present study aims to evaluate the immunoexpression profile of tumor microenvironment markers in the process and carcinogenesis of vermilion lip carcinoma.

## Methods

### Ethical considerations

The Research Ethics Committee approved the Haroldo Juaçaba Hospital/Ceará Cancer Institute research under registration protocol number 2.191.839. The study complies with the norms that regulate research in human beings, as stated in resolution 466/12 of the National Health Council. Furthermore, the study was conducted using the scientific methodology known as the STROBE initiative.

### Sample calculation and study groups

**Figure 1:**
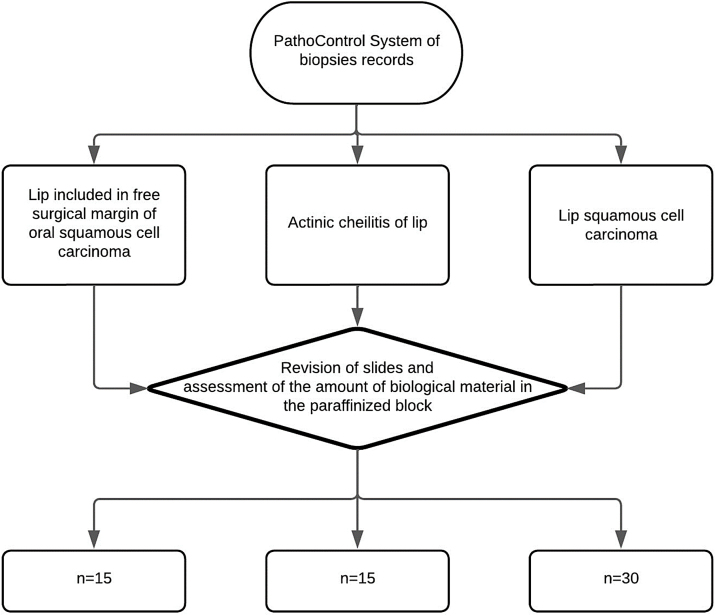
Flowchart of sample selection from laboratories without histological changes, actinic cheilitis and SCC from laboratories.

The sample will consist of 30 Squamous Cell Carcinoma of the lip, 15 actinic cheilitis and 15 lips without pathological changes according to the sample calculation performed based on the study by Kakasheva-mazhenkovska et al.^9^ This work showed that the histological grade of Squamous Cell Carcinoma of the lip is important for tissue invasion through immunoexpression for lesions that present a greater vascular density in more intense histological grades (20.37 ± 4.36 vs. 24.90 ± 6.27). Thus, adopting a case-to-control ratio of 2:1, it is necessary to evaluate 30 Squamous Cell Carcinoma of the lip and 15 control tissues (15 actinic cheilitis and 15 without pathological alteration) in order to obtain a sample that represents 80% of power and 95% confidence the alternative hypothesis of this study (Kesley's method) (Fig. 1).

### Inclusion and exclusion criteria

Inclusion criteria was: paraffinized blocks from incisional and excisional biopsies of patients treated at Hospital Haroldo Juaçaba, Instituto do Câncer do Ceará. After reviewing the amount of material available in the paraffinized block and patients' records, blocks without enough material and patients thar did not have clinical information were excluded (Patients whose paraffinized blocks came from review of histological slides).

### Data collection

For the present study, samples were collected from patients diagnosed and treated at the Hospital Haroldo Juaçaba, Instituto do Câncer do Ceará from 2015‒2020 who underwent surgery for lip vermilion carcinoma resection and did not undergo neoadjuvant treatment.

Tissues from healthy lip epithelia from head and neck squamous cell carcinoma (control group) were resected. Head and neck tumors that showed resection of lip were histologically analyzed, and paraffin blocks and histological slides with samples of lips from deep mucoceles.^10^ Additionally, tissues from Aquitic Cheilitis and Squamous Cell Carcinoma of the Lip cases from the Pathology Laboratory of Hospital Haroldo Juaçaba, Instituto do Câncer do Ceará were selected.

After revision of the slides, samples of the vermilion epithelium of the lip, free of tumor margins, from head and neck surgical resections were included in the control group. Patients with a revised histological diagnosis of Aquitic Cheilitis were included in the Aquitic Cheilitis group. Cases with a revised histological diagnosis of Squamous Cell Carcinoma were included in the Squamous Cell Carcinoma of the Lip group.

Clinicopathological data were collected from the patients' medical records, excluding patients with incomplete records (>30% of the sociodemographic and clinical-prognostic information). After evaluating the paraffin blocks, we excluded the blocks that did not present enough material to perform the Tissue Microarray (TMA) technique.

Histological, formalin-fixed, and paraffin-embedded specimens were selected, and the demographic and clinical data retrieved from the patients' medical records were analyzed. Histological slides of the excisional biopsies were evaluated and identified to fabricate the TMA blocks.

### Histological grading of LSCC and actinic cheilitis

The classification of A Aquitic Cheilitis and Squamous Cell Carcinoma lips was performed by an experienced pathologist (>10-years of oral pathology), with an intra-examiner calibration coefficient kappa = 0.859.

The Aquitic Cheilitis had their epithelial dysplasia classified using a binary system of low/high risk of malignant transformation.^11^ The Squamous Cell Carcinoma lips samples were classified using Bryne's binary model, classifying Squamous Cell Carcinoma into high-and low-grade.^12^ The presence of perineural and vascular invasion was also assessed.^13^

### TMA and immunohistochemistry

All microscopic slides were analyzed, and sites of tumors with highly cellular Squamous Cell Carcinoma lips sections, areas of Aquitic Cheilitis showing epithelium and connective tissue with solar elastosis, and control areas showing epithelium and connective tissue were identified. We selected two representative circumferential areas, each 2 mm in diameter (3.14 mm^2^) from each sample, for the microarray tissue technique. Paraffin blocks were punched from each demarcated area using a tissue microarrayer (Quick-Ray UNITMA®, Seongnam-si, South Korea) and then transferred to a paraffin receptor block containing 70 circular wells of the same diameter (2 mm). The paraffin receptor blocks containing the oral ulcers were then cut into 3-µm thick sections placed on silanized slides.

For immunohistochemical processing, samples were deparaffinized, rehydrated, and subjected to antigen retrieval in Tris-EDTA buffer (Ph 9.0). Samples were incubated in Phosphate-Buffered Saline (PBS) with 3% H_2_O_2_ for 30 min to inactivate endogenous peroxidases. Then, they were washed in PBS and incubated for one h with primary antibodies against CD3 (Dako^®️^, A0452), CD8 (Dako^®️^, M710), CD20 (Dako^®️^, m0755), CD68 (Dako^®️^, M0876) and ki-67 (Dako^®️^, MIB-1) was also used.

Samples were washed in PBS, incubated in Envision Plus HRP Anti-IgG-rabbit/mouse for 30 min (ready-to-use; monoclonal; Dako® K4065), and washed again in PBS, after which diaminobenzidine chromogen (Dako® K3469) was applied to the samples for 5 min. Harris hematoxylin was used as the counterstain (10 s), after which the specimens were dehydrated in ethanol and xylene and covered with a permanent mounting medium (Enthelam®). Colorectal carcinoma sections were used as a positive control, and the negative control and positive control were treated in parallel with an antibody diluent instead of the primary antibody.

### Immunohistochemical evaluation

For evaluation of CD3, CD8, CD20, and CD68, ten fields per histological section intraepithelial (normal lip mucosa or Aquitic Cheilitis) and intratumoral (lip cancer and Squamous Cell Carcinoma) or subepithelial (normal lip mucosa or Aquitic Cheilitis) and peritumoral (lip cancer and Squamous Cell Carcinoma) were photographed at 400× magnification. The images were exported to ImageJ® software and counted to determine the number of immunostained positive cells (brown staining stromal cells). For evaluation of ki-67, ten fields per histological section were photographed at 400× magnification, and brown nuclear staining of epithelial cells (control group and AC group) and cancer cells (lip cancer and Squamous Cell Carcinoma group) was considered a positive immunoreaction.^12^ The images were also exported to ImageJ® software and counted to determine the percentage of immunostained positive cells.

### Statistical analysis

Data were tabulated in Microsoft Excel (Microsoft Corporation®) and exported to the Statistical Package for the Social Sciences (SPSS) software, in which the analyses were performed with a 95% confidence level.

The mean and standard deviation of the percentages of immunostaining for each protein were calculated. These data were analyzed using the Kolmogorov-Smirnov normality test and did not have a Gaussian distribution. Therefore, the comparisons were performed using Mann-Whitney and Kruskal-Wallis or Dunn tests (between-group analysis).

## Results

### Sample characterization and immunostaining profile

The sample consisted of 15 lip mucosal epithelia without microscopic changes, 30 AC, and 45 lip cancer and Squamous Cell Carcinoma. Most Aquitic Cheilitis had low-risk dysplasia (*n* = 19), and the Lip Cancer and Squamous Cell Carcinoma was low-grade (*n* = 29). Only 5 lip cancer and Squamous Cell Carcinoma samples had lymphovascular invasion, and 13 had a perineural invasion.

**Figure 2:**
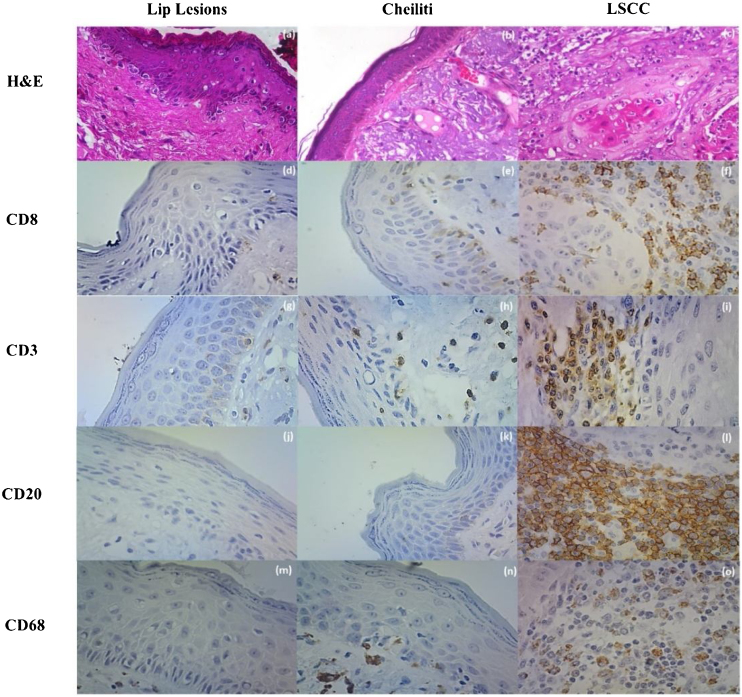
(a) Lip Lesions on HE stain; (b) Cheiliti on HE stain; (c) LSCC in HE stain; (d) Immunohistochemistry for CD8 in Lip Lesions; (e) Immunohistochemistry for CD8 in Cheiliti; (f) Immunohistochemistry for CD8 in LSCC; (g) Immunohistochemistry for CD3 in Lip Lesions; (h) Immunohistochemistry for CD3 in Cheilitis; (i) Immunohistochemistry for CD3 in LSCC; (j) Immunohistochemistry for CD20 in Lip Lesions; (k) Immunohistochemistry for CD20 in Cheiliti; (l) Immunohistochemistry for CD20 on LSCC; (m) Immunohistochemistry for CD68 on Lip Lesions; (n) Immunohistochemistry for CD68 in Cheilitis; (o) Immunohistochemistry for CD68 on LSCC.

There were CD3+, CD8+, and CD68+ cells in the lip epithelia without microscopic changes, but no CD20+ cells were observed. These cells were sparse among the epithelial cells without a specific topographic distribution. In the Aquitic Cheilitis, there was no significant increase in any of these cellular elements intraepithelial. Still, in the Lip Cancer and Squamous Cell Carcinoma, there was an increase in the amount of CD3+ (*p* < 0.001), CD8+ (*p* = 0.035), CD20+ (*p* < 0.001) and CD68+ (*p* < 0.001) cells within the tumor islands (intratumoral) (Table 1, Fig. 2).

**Table 1:** Immunostaining profile for inflammatory stromal cells Peritumoral (PT) and Intratumoral (IT) in the removed lip lesions and influence of microscopic findings and histological gradation on the immunolabeling profile.

	CD3		CD8		CD20		CD68	
	PT	IT	PT	IT	PT	IT	PT	IT
Lip lesions								
Lip (*n* = 15)	129.5 ± 57.2	38.9 ± 23.2	26.8 ± 19.4	12.3 ± 7.8	5.1 ± 10.2	0.0 ± 0.0	54.9 ± 52.1	1.3 ± 4.0
Cheilitis (*n* = 30)	138.2 ± 124.8	21.1 ± 17.0	43.4 ± 36.5^c^	14.6 ± 22.1	7.4 ± 15.8	0.0 ± 0.0	81.8 ± 69.1^c^	0.9 ± 2.9
LSCC (*n* = 45)	212.8 ± 135.3^b^, ^c^	67.3 ± 41.8 b,	128.5 ± 96.1^b^, ^c^	48.2 ± 48.9^b^, ^c^	85.7 ± 86.7^b^, ^c^	9.4 ± 11.0^b^, ^c^	31.1 ± 24.7^b^, ^c^	53.9 ± 33.4^b^, ^c^
*p*-value^a^	**0.037**	**<0.001**	**<0.001**	**0.035**	**<0.001**	**<0.001**	**0.001**	**<0.001**
Histological grading of dysplasias (Cheilitis)								
Low risk (*n* = 19)	145.5 ± 136.9	24.1 ± 19.3	46.8 ± 42.7	18.9 ± 26.4	7.0 ± 17.3	0.0 ± 0.0	94.9 ± 78.0	0.4 ± 0.5
High risk (*n* = 11)	122.5 ± 103.1	14.7 ± 8.6	36.7 ± 21.1	6.0 ± 3.6	7.9 ± 15.1	0.0 ± 0.0	61.1 ± 50.8	1.7 ± 4.5
*p*-value^a^	0.721	0.275	0.596	0.366	0.914	1.000	0.327	0.372
Histological grading of LSCC								
Low (*n* = 29)	233.4 ± 152.6	71.0 ± 45.6	142.7 ± 112.9	46.7 ± 42.1	90.5 ± 85.0	12.6 ± 11.6^b^	32.7 ± 30.2	52.2 ± 36.5
High (*n* = 17)	173.3 ± 96.6	62.4 ± 36.9	108.3 ± 56.7	53.2 ± 62.3	68.2 ± 88.9	3.8 ± 7.5	29.8 ± 8.1	57.3 ± 29.2
*p*-value^a^	0.179	0.532	0.314	0.705	0.460	**0.019**	0.755	0.675
Lymphovascular invasion (LSCC)								
No (*n* = 40)	207.9 ± 138.9	68.8 ± 44.5	132.3 ± 103.0	50.1 ± 52.6	82.8 ± 82.2	10.6 ± 11.5	31.2 ± 26.6	54.8 ± 34.9
Yes (*n* = 5)	236.8 ± 130.3	59.4 ± 19.4	117.8 ± 49.3	41.8 ± 19.7	81.4 ± 117.9	2.4 ± 3.4	34.6 ± 8.8	48.6 ± 29.2
*p*-value^a^	0.663	0.645	0.762	0.732	0.973	0.126	0.781	0.709
Perineural invasion (LSCC)								
No (*n* = 32)	220.1 ± 144.0	66.4 ± 42.9	130.5 ± 99.5	50.4 ± 55.8	92.2 ± 85.2	11.6 ± 12.0^c^	31.4 ± 28.0	55.2 ± 35.1
Yes (*n* = 13)	190.3 ± 120.3	71.1 ± 42.2	129.5 ± 95.2	45.0 ± 27.0	56.8 ± 86.4	3.7 ± 4.6	32.4 ± 12.6	50.3 ± 31.7
*p*-value^a^	0.532	0.748	0.978	0.770	0.271	**0.026**	0.917	0.716

a Test Kruskal-Wallis/Dunn.

b *p* < 0.05 versus lip.

c *p* < 0.05 versus cheilitis (media ± SD).

In the subepithelial tissue of the lip epithelia without microscopic changes most of the cells were CD3+ cells, but CD8+, CD68+ and to a lesser extent CD20+ cells could also be identified. The Aquitic Cheilitis showed a significant increase of CD8+ (*p* < 0.001) and CD68+ (*p* < 0.001) intraepithelial cells, but not CD3+ or CD20+ cells (*p* > 0.05). In lip cancer and Squamous Cell Carcinoma there was peritumoral increase in CD3+ (*p* = 0.037), CD8+ (*p* < 0.001), as well as CD20+ (*p* < 0.001) and CD68+ (*p* < 0.001) cells (Table 1).

Dysplasia gradation and lymphovascular invasion showed no significant association with the immunoexpression of any of the markers studied. Still, low-grade lip cancer and Squamous Cell Carcinoma (*p* = 0.019) and without perineural invasion (*p* = 0.026) showed higher intratumoral CD20+ cell immunoexpression (Table 1).

### The tumor microenvironment did not play a role in the clinical profile of LSCC, but cells interact significantly amongst

Among the 45 cases of lip cancer and Squamous Cell Carcinoma the majority were men (*n* = 34), aged less than 65 years (*n* = 24), T1/2 tumors (*n* = 30), N0 (*n* = 29), M0 (*n* = 47) and free of recurrence in five years (*n* = 47). The only clinical feature that showed an association with the immunoexpression of one of the markers was nodal metastasis. (Table 2).

**Table 2:** Influence of clinical features on the Immunostaining profile for inflammatory stromal cells peritumoral (PT) and intratumoral (IT) in the LSCC.

	CD3		CD8		CD20		CD68	
	PT	IT	PT	IT	PT	IT	PT	IT
Sex								
Female (*n* = 11)	192.3 ± 138.3	46.1 ± 30.8	98.6 ± 99.3	36.7 ± 45.3	55.3 ± 88.8	5.5 ± 11.6	63.3 ± 60.7	21.5 ± 31.5
Male (*n* = 34)	174.8 ± 124.6	52.9 ± 43.2	92.5 ± 85.0	38.6 ± 45.5	49.6 ± 71.2	5.1 ± 8.3	42.6 ± 43.7	34.5 ± 37.9
*p*-value^a^	0.594	0.498	0.807	0.886	0.780	0.889	0.120	0.177
Age								
Up to 65 (*n* = 24)	175.2 ± 111.0	53.3 ± 42.9	105.8 ± 101.1	42.0 ± 43.4	59.1 ± 76.3	7.4 ± 10.8	42.5 ± 38.3	33.4 ± 35.1
>65 (*n* = 21)	186.2 ± 146.9	47.7 ± 35.7	82.4 ± 74.3	34.1 ± 47.0	44.3 ± 77.1	3.2 ± 7.5	56.0 ± 59.7	27.2 ± 37.6
*p*-value^a^	0.718	0.550	0.314	0.529	0.432	0.069	0.276	0.490
T								
T1/2 (*n* = 30)	182.5 ± 117.3	52.3 ± 33.8	153.1 ± 116.1	63.3 ± 59.1	94.1 ± 78.4	15.5 ± 13.3	27.1 ± 17.7	46.9 ± 32.3
T3/4 (*n* = 15)	190.0 ± 142.5	70.2 ± 40.1	165.0 ± 162.3	36.4 ± 22.2	76.8 ± 108.8	11.4 ± 17.0	51.0 ± 48.9	53.4 ± 39.1
*p*-value^a^	0.915	0.379	0.872	0.350	0.728	0.615	0.181	0.737
N								
N0 (*n* = 29)	203.3 ± 109.4	67.3 ± 35.7	179.8 ± 131.9	65.3 ± 61.6	117.3 ± 97.6	13.0 ± 12.6	24.1 ± 13.3	56.9 ± 34.0
N+ (*n* = 16)	148.3 ± 124.6	59.5 ± 46.5	123.0 ± 123.5	37.8 ± 23.3	44.8 ± 41.3	15.8 ± 17.3	51.5 ± 45.1	37.3 ± 31.8
*p-*value^a^	0.360	0.705	0.418	0.320	0.112	0.719	0.105	0.284
M								
M0 (*n* = 47)	207.5 ± 128.7	67.3 ± 37.2	168.4 ± 133.1	66.2 ± 57.7	89.9 ± 82.1	16.8 ± 14.0	37.5 ± 37.5	54.4 ± 33.4
M1 (*n* = 3)	108.3 ± 67.1	48.0 ± 33.1	57.7 ± 28.4	35.0 ± 26.7	56.3 ± 39.3	7.7 ± 8.0	20.0 ± 14.5	23.3 ± 13.6
*p*-value^a^	0.235	0.439	0.192	0.394	0.517	0.313	0.457	0.154
Recurrence								
No (*n* = 47)	221.2 ± 136.0	69.6 ± 41.8	131.6 ± 97.9	49.5 ± 50.4	88.3 ± 89.4	9.0 ± 10.5	30.9 ± 25.0	56.4 ± 33.4
Yes (*n* = 3)	104.7 ± 69.3	37.0 ± 35.0	94.0 ± 78.9	32.3 ± 25.7	54.3 ± 37.6	13.7 ± 18.0	33.0 ± 26.1	26.3 ± 18.8
*p*-value^a^	0.153	0.197	0.524	0.566	0.522	0.487	0.889	0.137

*p <  0.05 (mean ± SD).

a Mann-Whitney head.

In lip epithelia without microscopic changes there was only a positive correlation between subepithelial immunoexpression of CD68+ and CD3+ cells (*p* =  0.002, *r* = 0.883). In the subepithelial region of the Aquitic Cheilitis CD3+ and CD8+ cells showed significant direct correlation (*p* =  0.035, *r* = 0.547), as well as CD68+ and CD3+ cells (*p* =  0.008, *r* = 0.637) and CD8+ cells (*p* =  0.017, *r* = 0.625) (Table 3).

**Table 3:** Correlation between inflammatory stromal cells Peritumoral (PT) and Intratumoral (IT) in the removed lesions of the lip.

		PT^a^				IT^a^			
		CD3	CD8	CD20	CD68	CD3	CD8	CD20	CD68
Lip									
CD3	*r*	‒	0.019	0.400	*0.883	‒	0.982	0.000	−0.072
	*p*-value	‒	0.975	0.252	**0.002**	‒	0.018	1.000	0.843
CD8	*r*	‒	‒	0.880	−0.567	‒	‒	0.000	0.132
	*p*-value	‒	‒	0.120	0.433	‒	‒	1.000	0.868
CD20	*r*	‒	‒	‒	0.556	‒	‒	‒	0.000
	*p*-value	‒	‒	‒	0.076	‒	‒	‒	1.000
CD68	*r*	‒	‒	‒	‒	‒	‒	‒	‒
	*p*-value	‒	‒	‒	‒	‒	‒	‒	‒
ki67	*r*	‒	‒	‒	‒	‒	‒	‒	‒
	*p-*value	‒	‒	‒	‒	‒	‒	‒	‒
Queilitis									
CD3	*r*	‒	*0.547	0.386	*0.637	–	−0.075	0.000	0.133
	*p*-value	‒	**0.035**	0.193	**0.008**	–	0.848	1.000	0.635
CD8	*r*	‒	–	0.396	*0.624	–	–	0.000	−0.072
	*p-*value	‒	–	0.203	**0.017**	–	–	1.000	0.865
CD20	*r*	‒	–	–	−0.185	–	–	–	0.000
	*p*-value	‒	–	–	0.526	–	–	–	1.000
CD68	*r*	‒	–	–	–	–	–	–	–
	*p*-value	‒	–	–	–	–	–	–	–
ki67	*r*	‒	–	–	–	–	–	–	–
	*p*-value	‒	–	–	–	–	–	–	–
LSCC									
CD3	*r*	‒	*0.467	0.288	0.028	‒	0.266	0.220	0.121
	*p*-value	‒	**0.004**	0.084	0.872	‒	0.112	0.190	0.487
CD8	*r*	‒	‒	*0.390	0.185	‒	‒	*0.396	*0.411
	*p*-value	‒	‒	**0.017**	0.288	‒	‒	**0.014**	**0.013**
CD20	*r*	‒	‒	‒	−0.177	‒	‒	‒	0.204
	*p*-value	‒	‒	‒	0.301	‒	‒	‒	0.234
CD68	*r*	‒	‒	‒	‒	‒	‒	‒	‒
	*p*-value	‒	‒	‒	‒	‒	‒	‒	‒
ki67	*r*	‒	‒	‒	‒	‒	‒	‒	‒
	*p-*value	‒	‒	‒	‒	‒	‒	‒	‒

a Spearman correlation.

* *p* < 0.05 versus other group (mean ± SD)

In peritumoral lip cancer and Squamous Cell Carcinoma, CD3+ and CD8+ cells showed a significant direct correlation (*p* =  0.004, *r* = 0.467), as well as CD68+ and CD8+ cells (*p* =  0.017, *r* = 0.390). In the intratumoral region CD8+ cells showed a significant correlation with CD20+ (*p* =  0.014, *r* = 0.396) and CD68+ (*p* =  0.013, *r* = 0.411) cells (Table 3).

There were no correlations between the immunoexpression of any intra- and subepithelial markers of the lip epithelia without microscopic changes. In the Aquitic Cheilitis, the immunoexpression of CD3 (*p* <  0.001, *r* = 0.878) and CD8 (*p* =  0.025, *r* = 0.641) showed a direct correlation between the intraepithelial and subepithelial regions. In lip cancer and Squamous Cell Carcinoma the immunoexpression of CD3+ (*p* =  0.002, *r* = 0.474), CD8+ (*p* =  0.001, *r* = 0.521) and CD68+ (*p* =  0.030, *r* = 0.363) showed direct correlation between intra and peritumoral region (Table 4).

**Table 4:** Correlation between Peritumoral (PT) and Intratumoral (IT) inflammatory stromal cells in the removed lesions of the lip.

	IT vs. PT			
	CD3^a^	CD8^a^	CD20^a^	CD68^a^
Lip				
r	0.551	−0.498	0.000	0.086
*p*-value	0.099	0.502	1.000	0.791
Queilitis				
r	*0.871	*0.641	0.000	0.320
*p*-value	**<0.001**	**0.025**	1.000	0.211
LSCC				
r	*0.474	*0.521	0.265	*0.363
*p*-value	**0.002**	**0.001**	0.107	**0.030**

a Spearman correlation.

* *p* < 0.05 versus other group (mean ± SD)

## Discussion

Tumor Microenvironment has been frankly studied in several tumors and head and neck cancers, playing an immunological role, where cells of the immune system have shown significant association with prognosis. Tumor Microenvironment may induce a key role in cancer progression and resistance to treatment in the tumor. The number of infiltrated immune cells may reflect in the antitumor immune response, and thus the tumor microenvironment becomes a possible predictor of treatment resistance, prognosis, and overall survival.^14^, ^15^

Our study evaluated the immunoexpression of CD3, CD8, CD20 and CD68 at different stages of lip cancer and Squamous Cell Carcinoma progression. We observed an increase of all these elements, especially around the tumor and within the tumor. Previous studies show that cancer in general, including Head and Neck Squamous Cell Carcinoma, tend to increase its inflammatory profile, including immune cell profile, to create a pathologically favorable microenvironment contributing to tumor progression,^16^, ^17^ which justifies one of the findings in our study.

We observed a significant increase in the expression of CD68 in the peritumoral region in Aquitic Cheilitis. This increase may be related to a migration of phagocytic mononuclear cells (macrophages) into the tumor according to its progression in an attempt to contain it.^18^ The rise in macrophages induces the overproduction of pro-inflammatory cytokines such as IL-1 and IFN that eventually stimulate the migration and activation of CD8 lymphocytes.^19^, ^20^

CD20 immunoexpression also showed a significant increase in the intratumoral region in lip cancer and Squamous Cell Carcinoma. Intriguingly, low-grade tumors were the primary responsible for this increase^21^ showed that increased CD20 expression is related to increased survival of patients with hematopoietic neoplasms and the presence of low-risk lesions. Distel et al.^22^ described a higher number of CD20+ B cells in early-stage squamous cell carcinoma of the hypopharynx associated with better loco-regional control. CD20+ cells are true producers of antibodies that play an essential role in humoral immunity against tumors. The antibodies can opsonize malignant cells and increase the cytotoxic activity of LTCD8 and, therefore, phagocytic activity of macrophages.^23^ Interestingly these three histological subtypes showed a significant correlation within tumors.

In our study, we observed a significant increase in the expression of CD3 and CD8 in peritumoral and intratumoral regions. As in other findings in the literature, where increased immunoexpression of these markers in the tumor microenvironment is related to an improvement in clinical progression, positively affecting the survival of patients with this neoplasm, generating an effective cytotoxic immune response.^10^, ^24^, ^25^

In contrast, the CD8/CD3 ratio was higher in vermilion lip carcinoma samples only in the peritumoral region, which shows that the amount of TCD8 lymphocytes concerning the universe of TCD3 lymphocytes was higher in the peritumoral region. However, for an effective cytotoxic effect of tumor cells to occur, it is necessary that tumor-infiltrating TCD8s bind FAS/FASL to induce cell apoptosisn.^26^ Conversely, CD8 can cause an antitumor response in the peritumor region. However, tumor cells can bypass death by preventing T-cell infiltration into the tumor.^25^

The microenvironment is an important component for cancer evasion. The same is composed by several cells in which they play a complex role in driving the Transition Epithelial-Mesenchymal (TME), in tumor progression and metastasis. In microenvironments tumor cells, the cells that support cancer cells such B lymphocytes, Lymphocytes T regulators and Tumor Infiltrantes Macrophages provide inhibit the epithelial state of the tumor, promote, and activate the state mesenchymal.^27^

In this context, these cells can also inhibit the migration and activity of anticancer immune cells, such as CD8+ T cells, NK cells and activated M1 macrophages. The cancer cells that reach the mesenchymal state can positively regulate the expression and activation of several cells of the immune system that influence tumor progression. Treg, M2 macrophages and B lymphocytes activated by cancer cells directly inhibit the function anticancer T-cells and NK cells to help tumor progression. These checkpoints play important role in oral squamous cell carcinoma progression and some checkpoints such PDL1, PD1 and CTLA4 are being used in immunotherapy with promising results, reinforcing the role of the tumor microenvironment in cancer progression.^28^

In Aquitic Cheilitis, we observed not only a relationship between CD68 with CD3 but of the stimulation of CD68 with CD8 and CD8 with CD3. This finding may be related to the initial changes in the microenvironment, which makes the site a more favorable environment for cell proliferation.^22^ In addition, the cells of potentially malignant lesions induce increased pro-inflammatory cytokines; thus, may present a conflict between pro-inflammatory mediators to eliminate dysplastic tissue against immunosuppressive cells that secrete molecules that can inhibit the immune system.^29^

## Conclusion

Despite the limitations of our study, we were able to plainly evaluate the transposition of Aquitic Cheilitis to lip cancer and Squamous Cell Carcinoma, showing the interaction of cells in the tumor microenvironment and noting that macrophage is the first interacting cell that has the greatest ability to migrate into the tumor and interact with CD3, CD8, and CD20. Apparently, CD20 impacts perineural invasion and histological gradation, having in more aggressive tumors lower amounts of CD20.

Nevertheless, given the complexity of the tumor microenvironment and the divergence of results among some studies, more studies addressing this issue are necessary to better understand the tumor microenvironment due to its importance for possible markers of prognosis survival and even potential immunological therapies.

## Funding

We thank the Fundação Cearense de Apoio ao Desenvolvimento Científico e Tecnológico (FUNCAP) in the form of a scientific initiation scholarship.

## Conflicts of interest

The authors declare no conflicts of interest.
